# Genome‐wide analysis of the AP2/ERF gene family in *Rheum officinale* Baill.: Evolution and expression profiling during plant development, abiotic stresses, and exogenous hormone responses

**DOI:** 10.1002/tpg2.70248

**Published:** 2026-05-11

**Authors:** Jing Tang, Gao‐chang Cui, Heng Pan, Hui Lu, Yi‐min Li, Feng Yan, Jing Gao, Liang Peng, Xiao‐chen Hu, Gang Zhang

**Affiliations:** ^1^ Key Laboratory for Research and Development of “Qin Medicine” of Shaanxi Administration of Traditional Chinese Medicine Shaanxi University of Chinese Medicine Xianyang China; ^2^ College of Pharmacy and Shaanxi Qinling Application Development and Engineering Center of Chinese Herbal Medicine Shaanxi University of Chinese Medicine Xianyang China; ^3^ State Key Laboratory of Research and Development of Characteristic Qin Medicine Resources (Cultivation) Shaanxi University of Chinese Medicine Xianyang China

## Abstract

The APETALA2/ethylene‐responsive factor (AP2/ERF) superfamily plays a central role in plant metabolism, stress responses, and hormone signaling. *Rheum officinale* Baill. is an important traditional medicinal plant whose roots and rhizomes are rich in anthraquinones and other secondary metabolites. However, the regulatory mechanisms underlying its development and secondary metabolism remain unclear, and systematic analyses of its AP2/ERF family are lacking. This study aimed to characterize the genomic features, expression patterns, and potential functions of the AP2/ERF family in *R. officinale*. A total of 167 RoAP2/ERF genes were identified, unevenly distributed across 11 chromosomes. Gene family expansion was mainly driven by segmental and tandem duplications, with extensive collinearity observed between *R. officinale* and related species. Phylogenetic, conserved domain, gene structure, and motif analyses classified these genes into five subfamilies (ERF, dehydration reaction element binding factor; AP2, related to abscisic acid insensitive 3/viviparous 1; and Soloist), with similar sequence characteristics within each subfamily. RNA‐seq analysis revealed tissue‐specific expression patterns, with Cluster 5 genes preferentially expressed in roots and rhizomes. RT‐qPCR of 18 representative genes confirmed their involvement in various signaling pathways. RoERF065 and RoERF079 were exclusively nuclear‐localized and strongly responsive to stress and hormone treatments. Functional assays indicated that RoERF079 acts as a C‐terminal‐dependent transcriptional activator, whereas RoERF065 may function as a repressor due to two EAR motifs. These genes may regulate root and rhizome development and secondary metabolism in *R. officinale*. This study provides a basis for elucidating the molecular mechanisms of organ development and bioactive compound biosynthesis and identifies candidate genes for molecular breeding.

## Plain Language Summary

The APETALA2/ethylene‐responsive factor (AP2/ERF) gene family helps plants grow, handle stress, and regulate hormone signaling. *Rheum officinale* is a medicinal plant, and its dried roots and rhizomes contain valuable anthraquinones. We identified 167 RoAP2/ERF genes in the genome, which were divided into five groups and expanded via gene duplication. Some of these genes are highly expressed in the roots and rhizomes. Among them, *RoERF065* and *RoERF079* respond to stress and hormone signals: one activates genes while the other suppresses them, which may play a role in plant growth and anthraquinone biosynthesis. This study helps in understanding these biological processes and identifying genes for improving plant breeding.

**Abbreviations**
ABA: abscisic acid
AP2/ERF: APETALA2/ethylene‐responsive factor
DREB: dehydration reaction element binding factor
ERF: ethylene‐responsive factor
Eth: ethylene
HT: high temperature
LT: low temperature
MeJA: methyl jasmonate
SA: salicylic acid

## 1 INTRODUCTION

*Rheum officinale* Baill., a perennial herb in the *Rheum* genus, is a key source of the traditional Chinese medicine Da Huang. The medicinal use of rhubarb involves dried roots and rhizomes, which are bitter and cold in nature. It is known for its purgative, heat‐clearing, detoxifying, blood‐stasis‐eliminating, menstruation‐regulating, and dampness‐removing effects (The Pharmacopoeia Commission of the People's Republic of China, 2020; Y. Wang et al., 2022). Anthraquinones, particularly rhein, emodin, aloe‐emodin, aloin, and chrysophanol, are the key bioactive compounds in rhubarb, responsible for its laxative, anti‐inflammatory, and antitumor effects (Hu et al., 2021; P. Li et al., 2017; Prateeksha et al., 2019). Despite extensive research on their bioactivity, the biosynthetic pathways of these anthraquinones remain unclear.

Plant growth, development, and secondary metabolite biosynthesis, key determinants of medicinal material quality, are orchestrated by transcription factors (TFs) via cis‐regulatory elements and modulation of metabolism and stress responses (Shi et al., 2016; Y. Zhang et al., 2018). The APETALA2/ethylene‐responsive factor (AP2/ERF) superfamily represents a major class of TFs regulating plant metabolism (Licausi et al., 2013). Members contain a conserved AP2 DNA‐binding domain of 60–70 amino acids (Jofuku et al., 1994). Based on domain architecture, the superfamily is classified into five subfamilies: AP2, ERF, dehydration reaction element binding factor (DREB), related to abscisic acid insensitive 3/viviparous 1 (RAV), and Soloist (Sakuma et al., 2002). The AP2 subfamily typically contains two AP2 domains (Shigyo & Ito, 2004), whereas RAV proteins contain both AP2 and B3 domains (Nakano et al., 2006), and Soloist proteins constitute a small (Sakuma et al., 2002), structurally distinct lineage. ERF and DREB groups are distinguished by conserved amino acid residues within the AP2 domain, particularly at positions 14 and 19, occupied by alanine/aspartic acid in ERF and valine/glutamic acid in DREB (Ohme‐Takagi & Shinshi, 1995).

Hormone‐ and stress‐responsive TFs, including WRKY, bHLH, bZIP, AP2/ERF, and NAC, are key regulators of secondary metabolism (Zheng et al., 2023). Among them, the AP2/ERF superfamily has been widely characterized across diverse plant species, including *Arabidopsis thaliana* (Nakano et al., 2006), *Myrica rubra* (Y. Liu et al., 2024), *Sesamum indicum* (Su et al., 2022), *Pyrus pyrifolia* (Xu et al., 2023), *Solanum melongena* (D. Li, He, et al., 2021), and *Limonium bicolor* (Jiang et al., 2024). These TFs have been shown to be involved in regulating multiple biological processes and metabolic pathways, such as biosynthesis of callus (Y. Zhang et al., 2024), flavonoid (C. Zhao et al., 2021), mannan (Zeng et al., 2025), terpenoid indole alkaloid (Paul et al., 2017), phenylpropanoid, and starch/sucrose (Liang et al., 2024). Additionally, they play crucial roles in plant growth and development, including flowering (Cheng et al., 2023), seed development (Jin et al., 2025), and root development (T. T. Zhang, Lin, et al., 2024), as well as stress tolerance (Wei et al., 2025). ERF/AP2 also mediate JA‐induced artemisinin and ethylene (Eth)‐induced anthocyanin biosynthesis (Ni et al., 2020; Yu et al., 2012). In *R. officinale*, recent studies have revealed the accumulation patterns of active constituents and the involvement of WRKY TFs, CYP450s, and UGTs in their biosynthesis (Y. M. Li et al., 2025). However, systematic characterization of AP2/ERF members remains limited, hindering understanding of its secondary metabolic regulatory network and molecular improvement of medicinal quality and stress adaptability.

This study performed a genome‐wide identification and systematic characterization of the AP2/ERF TF family in *R. officinale*. Gene structure, phylogenetic relationships, chromosomal localization, gene duplication, and cis‐acting elements were analyzed. RNA‐Seq and RT‐qPCR were used to examine tissue‐specific expression across developmental stages and under abiotic stresses and hormone treatments. Functional validation was conducted for two candidate genes, *RoERF065* and *RoERF079*. This study provides a foundation for elucidating AP2/ERF‐mediated regulation of anthraquinone biosynthesis and stress resistance in *R. officinale*.

## 2 MATERIALS AND METHODS

### 2.1 Gene identification and classification

Genomic data for *A. thaliana* were retrieved from The Arabidopsis Information Resource (TAIR; https://www.arabidopsis.org/). *Arabidopsis* AP2/ERF protein sequences were used to perform BLASTP searches against the full‐length protein sequences of *R. officinale* (*E*‐value < 1e−10). HMM profiles for the AP2 (PF00847) and B3 (PF02362) domains were obtained from the Pfam database (http://pfam.xfam.org/). A preliminary set of AP2/ERF genes was identified in the *R. officinale* genome using BLASTP and HMMER approaches (data from our in‐house genome project). The presence of AP2 and B3 domains was further validated using SMART (http://smart.embl‐heidelberg.de/) and the NCBI Conserved Domain Database (CDD; https://www.ncbi.nlm.nih.gov/Structure/cdd/wrpsb.cgi). After removing redundant sequences, 167 putative AP2/ERF genes were identified in the *R. officinale* genome for further analysis. The AP2/ERF protein sequences from *A. thaliana* and *R. officinale* are provided in Table S1. The AP2/ERF proteins’ basic properties were retrieved from ExPASy ProtParam (http://web.expasy.org/protparam/), subcellular localization was predicted using Cell‐PLoc 2.0 (http://www.csbio.sjtu.edu.cn/bioinf/plant‐multi/), and secondary structure was analyzed using SOPMA.

### 2.2 Phylogenetic tree analysis and classification of AP2/ERF genes

Multiple sequence alignments of full‐length AP2/ERF proteins from *R. officinale* and *A. thaliana* were performed using MUSCLE with default parameters. A phylogenetic tree was constructed using the neighbor‐joining method in MEGA 7 with 1,000 bootstrap replicates. The tree was visualized using the iTOL platform. *Rheum officinale* AP2/ERF proteins were classified into subgroups based on their phylogenetic relationships with Arabidopsis homologs.

### 2.3 Chromosomal distribution and duplication of RoAP2/ERF genes

Chromosomal locations of RoAP2/ERF genes were obtained from the *R. officinale* genome annotation. Gene duplication events were identified using MCScanX, including intra‐ and interspecies duplications involving *A. thaliana*, *Oryza sativa*, *Rheum tanguticum*, and *Rheum nobile*. Synonymous (Ks) and nonsynonymous (Ka) substitution rates of gene pairs were calculated using KaKs_Calculator 2.0.

### 2.4 Gene structure, conserved domains, motifs, and cis‐acting elements analysis

Conserved domains of RoAP2/ERFs were identified using the Batch CD‐Search tool (https://api‐www‐ncbi.laibokeji.com/Structure/bwrpsb/bwrpsb.cgi). Conserved motifs were identified using MEME. Cis‐acting elements in the 2000 bp upstream regions of each gene were analyzed using PlantCARE (http://bioinformatics.psb.ugent.be/webtools/plantcare/). Results were visualized using TBtools.

### 2.5 Plant materials, treatments, RNA sequencing, and data analysis

Seeds of *R. officinale* were collected in September 2023 from Zhenba County, Hanzhong City, Shaanxi Province, China. Seedlings were grown under controlled conditions (16 h light/8 h dark, 9,000 lux, 23 ± 2°C). Thirty‐day‐old seedlings were subjected to stress and exogenous hormone treatments. Leaves were sampled at 0, 2, 6, 12, and 24 h post‐treatment, with three biological replicates per treatment. Hormone concentrations and stress conditions are listed in Table S2.

To analyze tissue‐specific and stress‐responsive expression of RoAP2/ERF genes, FPKM data were retrieved from multiple transcriptome datasets, including different tissues (roots, stems, and leaves) of 2–4‐year‐old *R. officinale* (PRJNA1165988; Y. M. Li et al., 2025), methyl jasmonate (MeJA) treatment for 12 h (PRJNA1163520; Tang et al., 2024), temperature stress (12 h high‐ and low‐temperature treatments), and Eth treatment (2 h) (PRJNA1295681 and PRJNA1294498, unpublished). Total RNA was extracted from leaves of 30‐day‐old seedlings treated with 0.2 mM Eth (0 and 2 h) or exposed to high (40°C) or low (4°C) temperature stress (12 h), with three biological replicates per treatment. cDNA libraries were prepared and sequenced on the Illumina NovaSeq 6000 platform. Raw reads were filtered with fastp (v0.18.0), and clean reads were aligned to the long‐read transcriptome reference. Gene expression was quantified as FPKM using RSEM (v1.2.19). Differentially expressed genes were identified with DESeq2 (v1.20.0; |log_2_ fold change| > 1, *p* < 0.05), followed by GO and KEGG enrichment analyses. Log_2_‐transformed FPKM values were visualized as heatmaps using TBtools.

### 2.6 RNA extraction, quantitative RT‐PCR

Total RNA was extracted from *R. officinale* seedlings using a Plant RNA Rapid Extraction Kit (Vazyme, China). RNA quality was assessed using a NanoDrop spectrophotometer and an Agilent 2100 Bioanalyzer. cDNA was synthesized using the PrimeScript RT Master Mix kit (TaKaRa). Primers were designed using NCBI Primer‐BLAST, with *β*‐actin as the reference gene. Primer sequences are listed in Table S3. qPCR was performed using ChamQ SYBR qPCR Master Mix (Vazyme) on a StepOnePlus Real‐Time PCR System. Relative expression levels were calculated using the 2^−ΔΔCt method.

### 2.7 Co‐expression correlation analysis

Protein sequences of enzymes involved in the mevalonate (MVA), methylerythritol phosphate (MEP), shikimate, and polyketide pathways were retrieved from NCBI and used as BLASTP queries (*E*‐value < 1 × 10^−^^10^) against the *R. officinale* protein database. Candidate genes involved in anthraquinone biosynthesis were identified based on BLASTP results and conserved domain analysis (Tables S4–S5). Expression matrices of candidate enzyme genes and AP2/ERF genes were extracted from RNA‐seq FPKM data. Spearman correlation coefficients were calculated in R across treatments. Gene pairs with strong correlations (|*r*| ≥ 0.8, adjusted *p* < 0.01) were selected, and promoter structure analysis was subsequently performed to explore potential regulatory relationships between AP2/ERF TFs and anthraquinone biosynthesis.

### 2.8 Subcellular localization analysis of RoERF065 and RoERF079

Full‐length CDSs of ERF65 and ERF79 were cloned into the pCAMBIA1305 vector fused with EGFP. The constructs were introduced into *Agrobacterium tumefaciens* EHA105 and transiently expressed in tobacco leaves via syringe infiltration. Fluorescence signals were observed using confocal laser scanning microscopy. Primers are listed in Table S6.

### 2.9 Transcriptional activity analysis of RoERF065 and RoERF079

Recombinant plasmids pGBKT7‐RoERF065x and pGBKT7‐RoERF079x were constructed, where x indicates truncated fragments of different lengths. Constructs were transformed into Y2H Gold cells and plated on SD/‐Trp medium at 30°C for 24–72 h. Positive colonies were diluted and spotted onto SD/‐Trp/‐His/‐Ade medium with or without X‐α‐Gal. Plates were incubated at 30°C for 24–72 h and imaged. Primers are listed in Table S7.

### 2.10 Statistical analysis

RT‐qPCR data were analyzed using GraphPad Prism 10 and presented as mean ± SE (*n* = 3). Statistical significance was assessed by ANOVA using SPSS 26.0. A value of *p* < 0.05 was considered statistically significant.

## 3 RESULTS

### 3.1 Genome‐wide identification of AP2/ERF genes in *R. officinale* Baill

A total of 167 potential AP2/ERF genes were identified (Table 1; Table S8), each containing at least one AP2 domain. The CDS lengths of RoAP2/ERF genes ranged from 273 to 1968 bp, and the proteins consisted of 90 (RoERF037) to 655 (RoAP02‐07) amino acid residues. The molecular weight and isoelectric point varied considerably, ranging from 10,147.57 to 71,685.53 Da and 4.49 to 11.32, respectively. Analysis of the instability index revealed that only 21 RoAP2/ERF proteins were stable, while the remaining 146 were unstable. The aliphatic index of RoAP2/ERF proteins ranged from 41.62 to 100.34, and all members were hydrophilic. The secondary structure of RoAP2/ERF proteins was composed of *α‐*helices, *β*‐turns, random coils, and extended strands. Among these elements, alpha helices and random coils constitute the majority, averaging 19.58% and 73.88%, respectively, while extended strands represent a smaller proportion, averaging 6.53%. Predicted subcellular localizations indicated that 93 AP2/ERF proteins were nuclear, 62 were localized to the cytoplasm and nucleus, and 11 were exclusively cytoplasmic. The sole exception was RoRAV4, which was simultaneously localized to the chloroplast, cytoplasm, and nucleus.

**TABLE 1:** Comparison of the number of APETALA2/ethylene‐responsive factor (AP2/ERF) between *Arabidopsis thaliana* and *Rheum officinale*.

	*Arabidopsis thaliana*		*Rheum officinale*		
CLASSIFICATION	Group	Number	Group		Number
AP2 FAMILY	Total	18	Total		25
	Double AP2 domain	14	Double AP2 domain		21
	Single AP2 domain	4	Single AP2 domain		4
ERF FAMILY	Total	122	Total		132
DREB SUBFAMILY	A1–A6	57	A1–A6		53
ERF SUBFAMILY	B1–B6	65	B1–B6	More than two AP2 domains	5
				Single AP2 domain	74
RAV FAMILY	Single AP2 domain and B3 domain	6	Single AP2 domain		2
			Single AP2 domain and B3 domain		7
SOLOIST FAMILY	Single AP2 domain	1	Single AP2 domain		1

Abbreviations: DREB, dehydration reaction element binding factor; RAV, related to abscisic acid insensitive 3/viviparous 1.

### 3.2 Multiple sequence alignment, phylogenetic analysis, and classification of RoAP2/ERFs

**FIGURE 1:**
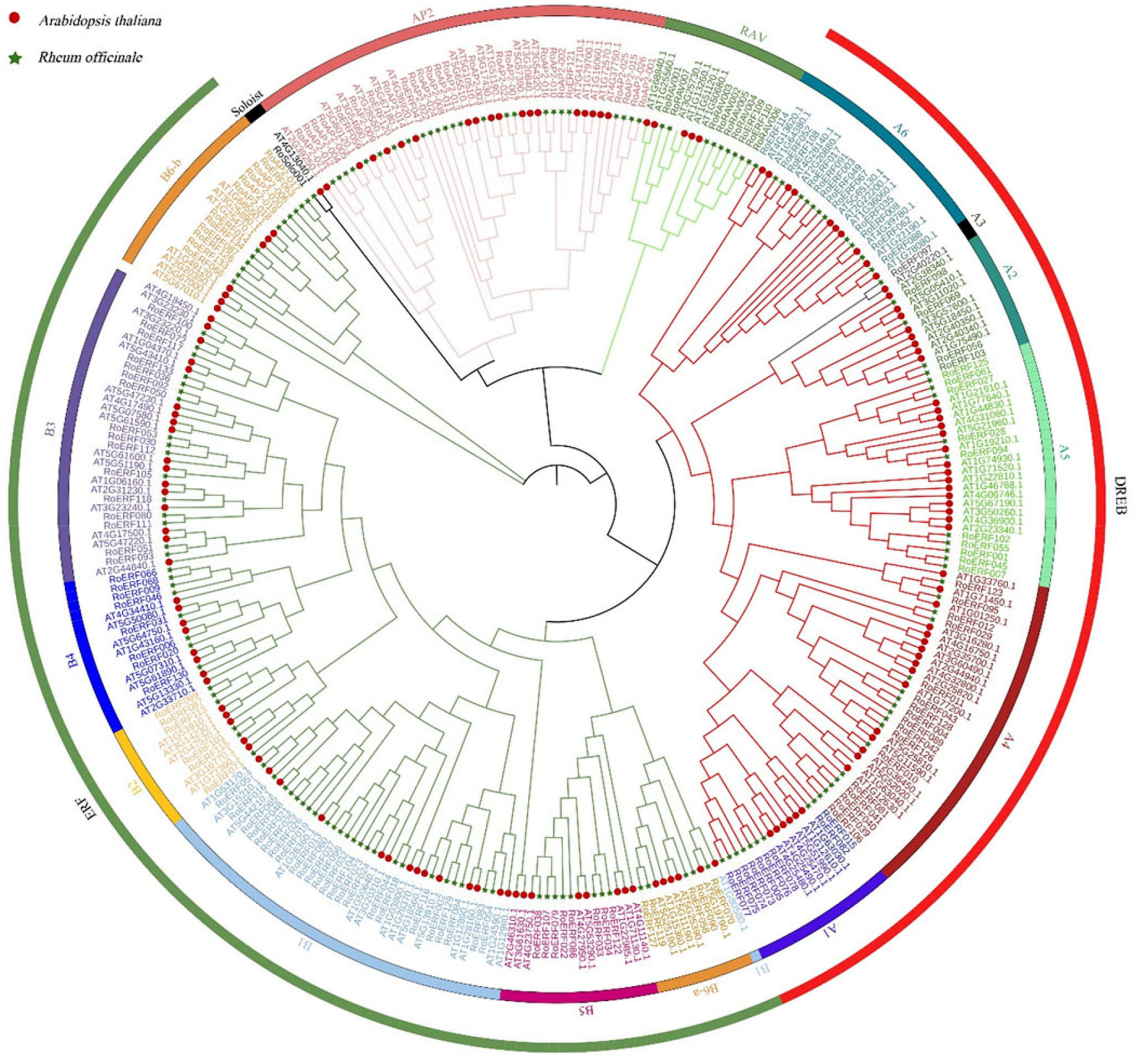
Neighbor‐joining phylogenetic tree of *Rheum officinale* and *Arabidopsis thaliana* APETALA2/ethylene‐responsive factor (AP2/ERF) family members. The AP2/ERF family members were divided and indicated in a distinct color.

**FIGURE 2:**
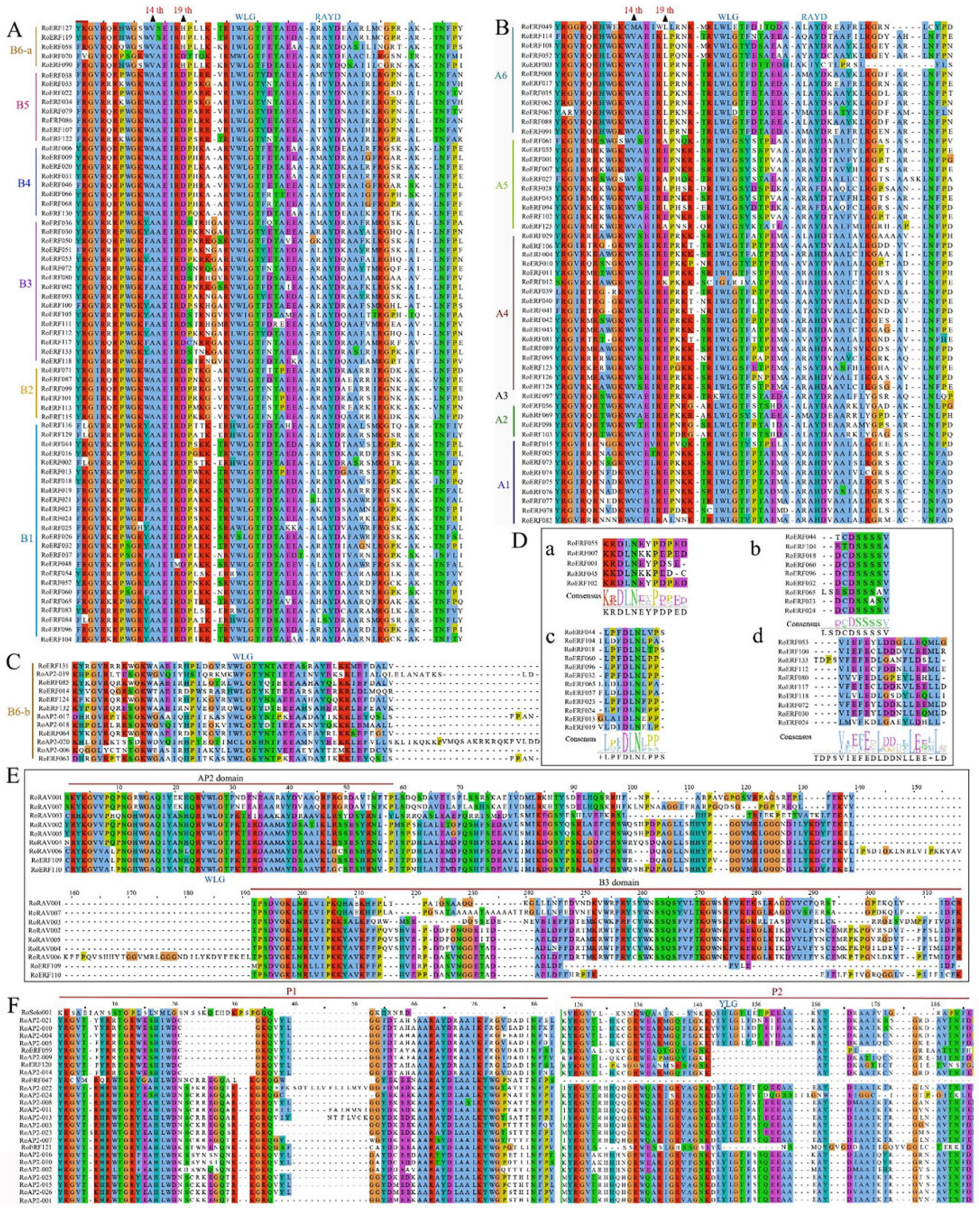
Multiple sequence alignment of conserved domains of APETALA2/ethylene‐responsive factor (AP2/ERF) proteins in *Rheum officinale*. (A) AP2 domain of the RoERF subfamily. (B) AP2 domain of the dehydration reaction element binding factor (DREB) subfamily. (C) AP2 domain of ERF members in subgroup B6‐b. (D) Other domains: (a) RoERF proteins in group A5 harbor the ERF‐associated amphiphilic repression (EAR) motif. (b) The DCDSSS motif of RoERF members in subgroup B1. (c) RoERF proteins in group B1 harbor the EAR motif. (d) The EDLL motif of group B3 RoERF proteins. (E) Conserved region of the related to abscisic acid insensitive 3/viviparous 1 (RAV) subfamily. (F) Conserved region of the AP2 subfamily. P1 and P2 denote the two AP2 domains respectively, with P1 located at the N‐terminal and P2 at the C‐terminal.

To explore the homologous relationship of AP2/ERFs in *R. officinale* and *A. thaliana*, we constructed a neighbor‐joining phylogenetic tree using 167 AP2/ERF and 147 AP2/ERF protein sequences, respectively (Figure 1). The results showed that RoAP2/ERFs could be divided into five subfamilies: ERF(B1–B6), DREB(A1–A6), AP2, RAV, and Soloist. Each subfamily includes both AtAP2/ERF and RoAP2/ERF proteins. The ERF subfamily was the largest, comprising 79 members, while the Soloist subfamily was the smallest, with only one member. Notably, four genes were assigned to the AP2 subfamily, two members to the RAV subfamily, and five members were placed in the B6‐b group of the ERF subfamily. These *RoAP2/ERFs* were systematically renamed based on the classification of the AP2/ERF superfamily and types of conserved domains (Table S8). Multiple sequence alignment revealed that all RoAP2/ERFs contained at least one AP2 domain (60–70 aa) (Figure 2). Most ERF and DREB proteins possessed the conserved WLG motif, whereas three RoERFs (RoERF013, RoERF050, RoERF068) exhibited a variant (G/W)L(G/E/R) motif. Distinct conserved motifs were observed among subgroups, including (L/F)DLNL/F(X)P in A5 and B1, DCDSSS in the N‐termini of B1, and EDLL in B3 proteins. The AEIR sequence (positions 14–17) was conserved in B1–B5 but replaced by SEIR in B6. AP2 subfamily proteins contained two conserved AP2 domains, whereas some ERFs (RoERF123, RoERF061, and RoERF122) retained only one, lacking the YLG motif. The Soloist subfamily showed substantial divergence in the AP2/ERF domain. RAV proteins contained both AP2 and B3 domains, with RoERF111 and RoERF112 exhibiting B3‐like sequences, supporting their classification (Figure 2).

### 3.3 Chromosomal distribution, gene duplication, and synteny analyses of AP2/ERF genes

**FIGURE 3:**
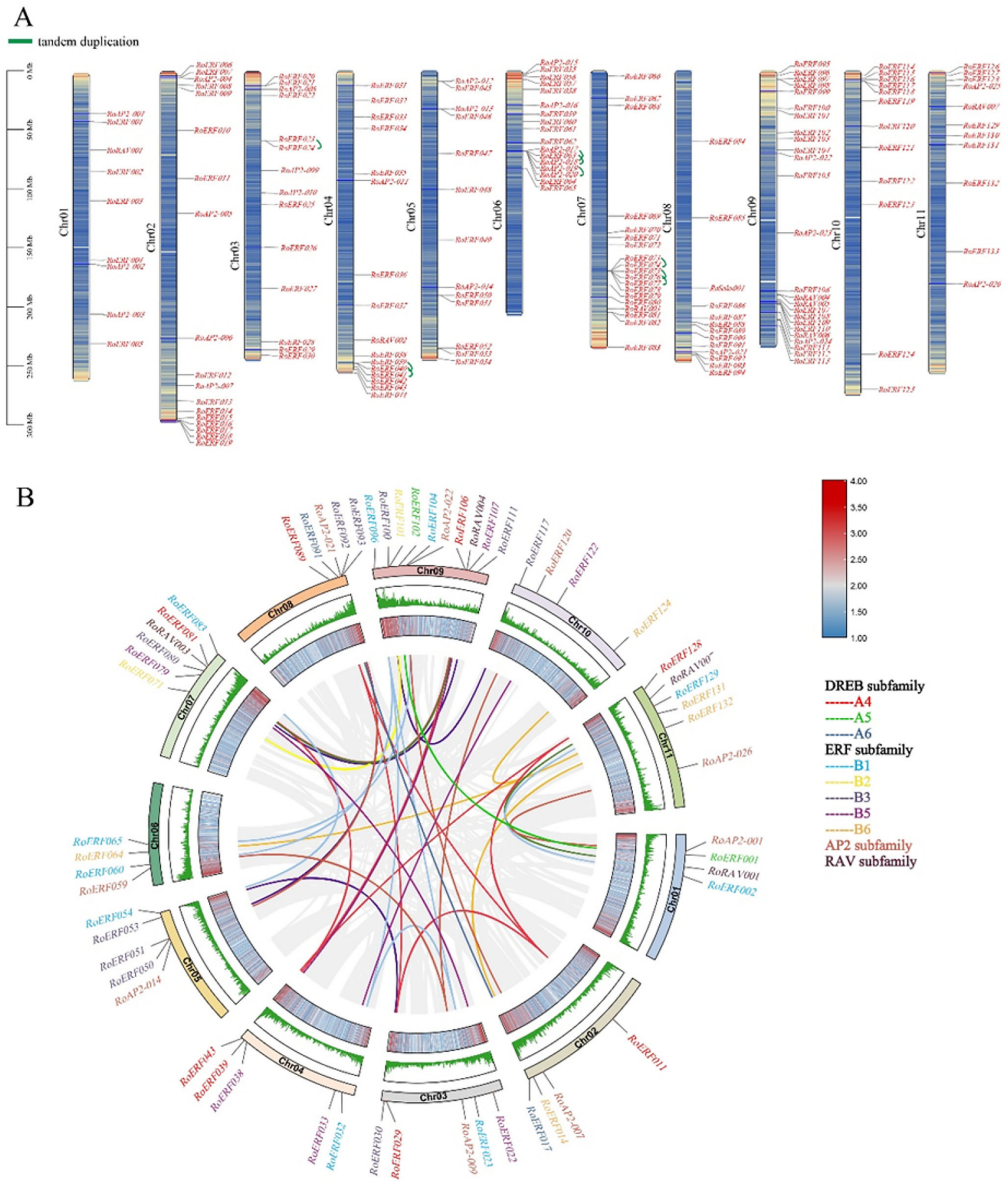
Chromosomal localization and synteny analysis of the APETALA2/ethylene‐responsive factor (AP2/ERF) genes in *Rheum officinale*. (A) Chromosomal locations of the RoAP2/ERF genes and schematic diagram of tandem gene pairs. (B) Synteny analysis of the RoAP2/ERF genes. The different colored lines in the middle represent the duplicated gene pairs of the AP2/ERF gene family in *R. officinale*.

**FIGURE 4:**
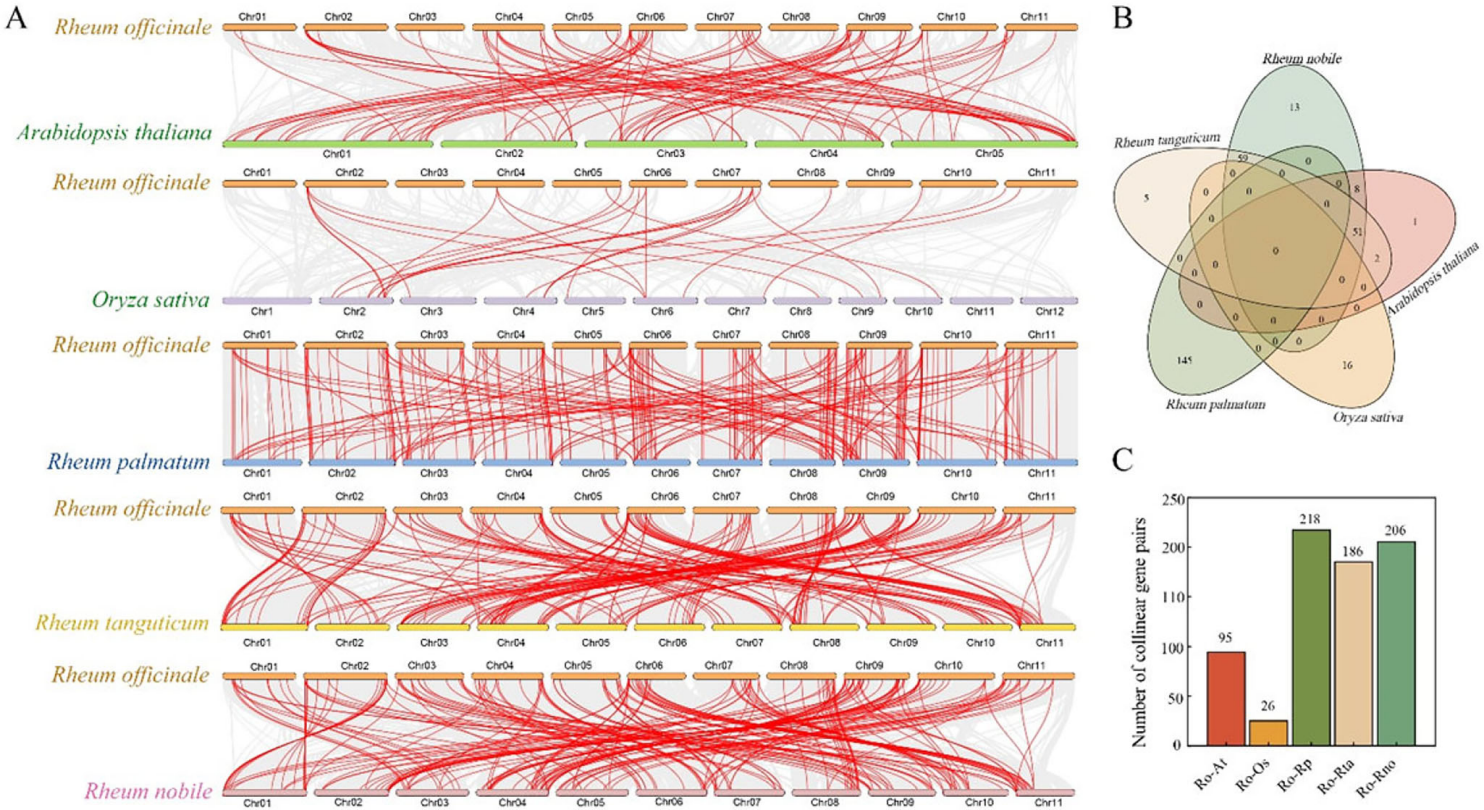
Distribution of homologous gene pairs of APETALA2/ethylene‐responsive factor (AP2/ERF) family members in *Rheum officinale*, *A. thaliana*, *O. sativa*, *R. tanguticum*, *R. palmatum*, and *R. nobile*. (A) Line segments represent chromosomes, numbers correspond to specific chromosome numbers, and red highlighted lines represent homologous AP2/ERF gene pairs between the two species. (B) The number of RoAP2/ERF genes collinear with different species. (C) The number of collinear gene pairs between RoAP2/ERF genes and genes of different species.

Chromosome mapping identified 167 AP2/ERF TFs unevenly distributed across 11 *R. officinale* chromosomes, with chromosome 09 harboring the most genes (25) and chromosome 01 the fewest (9) (Figure 3A). Synteny analysis revealed extensive duplication within the AP2/ERF superfamily, including 36 segmental or WGD pairs (58 genes) and nine tandem duplication events (18 genes) (Figure 3A,B; Table S9). Segmental duplication was most prevalent in subgroups B1 and B3 (10 genes each), followed by the AP2 subfamily (nine genes), whereas tandem duplication was confined to A1, A4, B1, and AP2. All collinear gene pairs exhibited Ka/Ks ratios < 1, indicating purifying selection and functional conservation (Table S9). Cross‐species synteny analyses showed that a total of 62 and 16 RoAP2/ERF genes formed 95 and 26 collinear pairs with *Arabidopsis* and rice, respectively (Figure 4; Tables S10–S13). In comparison with the three *Rheum* species, 218, 186, and 206 collinear pairs were identified, involving 145, 117, and 131 RoAP2/ERF genes, respectively. Notably, 145 RoAP2/ERF genes exhibited collinearity exclusively with *R. palmatum*, highlighting the closer evolutionary relationship between the *R. officinale AP2/ERF* gene set and that of *R. palmatum*.

### 3.4 Gene structure and motif composition of the RoAP2/ERF family

All RoAP2/ERFs contained at least one conserved AP2 domain (Figure S1). Five proteins (RoAP02‐06, RoAP02‐17, RoAP02‐18, RoAP02‐19, RoAP02‐20) harbored two to four AP2 domains but clustered within the ERF subfamily. DREB and Soloist members each contained a single AP2 domain, whereas RAV proteins possessed one AP2 and one B3 domain. RoERF109 and RoERF110 contained incomplete B3‐like motifs and were classified as RAV members. Four proteins (RoERF047, RoERF059, RoERF120, RoERF121) contained only one AP2 domain but were assigned to the AP2 subfamily. RoERF021, RoERF026, and RoERF104 harbored additional BAH, Chaperonin, and PLPDE‐III domains, respectively. Motif analysis revealed subfamily‐specific signatures (Figures S2–S3). ERF and DREB proteins shared motifs 1, 2, and 6. RAV proteins contained motifs 1, 4, 6, and 11, and additionally motifs 2, 14, and 20, except in RoERF109 and RoERF110. AP2 members possessed motifs 2, 3, 4, and 16, whereas Soloist proteins contained only motifs 1 and 6. Motifs 3 and 13 were specific to AP2, whereas motif 14 was unique to RAV. Gene structure analysis showed complementary insights into the evolutionary conservation and divergence of the RoAP2/ERF superfamily in *R. officinale* (Figure S4). AP2 genes were intron‐rich (2–9 introns), whereas DREB genes were mostly intronless. All RAV subfamily genes except RoERF110 lacked introns, and ERF genes contained 0–3 introns. The Soloist gene contained five introns. Genes within the same subfamily displayed similar intron patterns, indicating conserved evolution.

### 3.5 The putative promoter regions analysis of RoAP2/ERF genes

**FIGURE 5:**
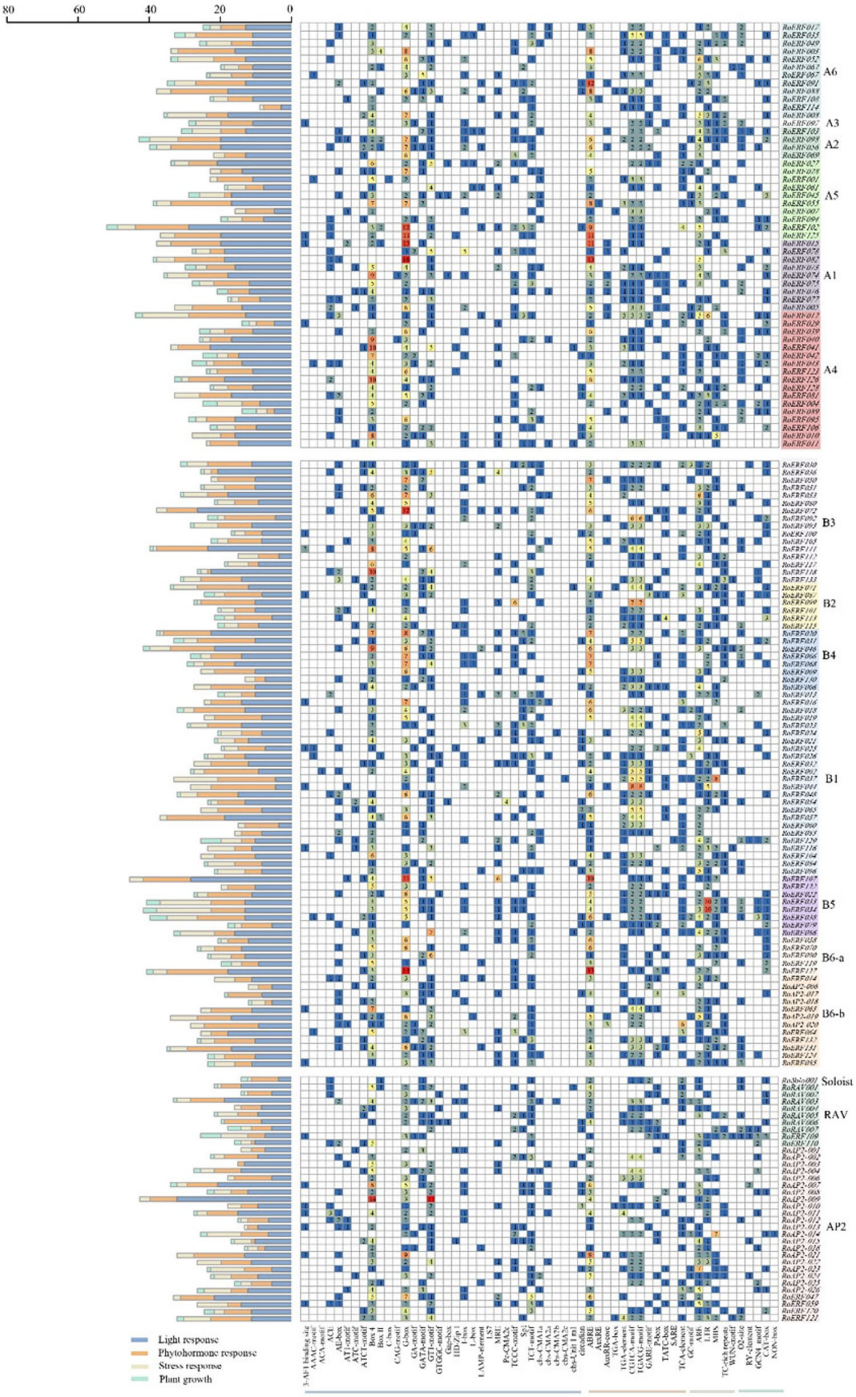
Analysis of cis‐acting elements of RoAP2/ERF (where AP2/ERF is APETALA2/ethylene‐responsive factor) genes and statistics of the number of four major types of response elements. The four types include phytohormone‐responsive, stress‐responsive, light‐responsive, and growth‐related elements.

To investigate the potential functions of the RoAP2/ERF gene family, 2000 bp upstream promoter regions of all 167 genes were analyzed for cis‐regulatory elements (Figure 5; Table S14). Identified elements were classified into four categories: light, hormone, growth and development, and stress responsiveness. Light‐responsive elements were ubiquitous, with Box 4 and G‐box being the most abundant. Hormone‐responsive elements were present in most promoters, predominantly ABRE and jasmonic acid‐responsive motifs (CGTCA and TGACG). Nearly all promoters, except RoERF076, contained stress‐related elements associated with anaerobic, drought, and low‐temperature responses. Eight genes harbored the wound‐responsive WUN motif. Additionally, 117 promoters contained developmental elements linked to meristem activity, seed‐specific expression, and cell cycle regulation.

### 3.6 Expression patterns of RoAP2/ERF genes across diverse plant tissues and their responses to heat, cold or UV‐B stress

Expression profiling across multiple tissues revealed strong tissue specificity of RoAP2/ERF genes (Figure S5; Table S15). After excluding 18 genes with no detectable expression in any sample, the remaining genes were classified into eight clusters based on hierarchical clustering. Cluster 7 genes were predominantly expressed in leaves, whereas Cluster 4 genes showed stable expression across tissues and developmental stages. Clusters 3 and 2 genes were enriched in roots of 3‐ and 4‐year‐old plants, respectively. Cluster 5 genes exhibited stable leaf expression but increased expression in roots and rhizomes with plant age. Under temperature stress, genes were classified into seven clusters (A–G) (Figure S5; Table S16). Cluster B genes were induced by high temperature (HT, 40°C), whereas Cluster D genes responded to low temperature (LT, 4°C). In contrast, Cluster C and Cluster G genes were downregulated by high and LT, respectively, while Cluster F genes showed opposite responses to high and LT. Cluster A genes showed no detectable expression.

**FIGURE 6:**
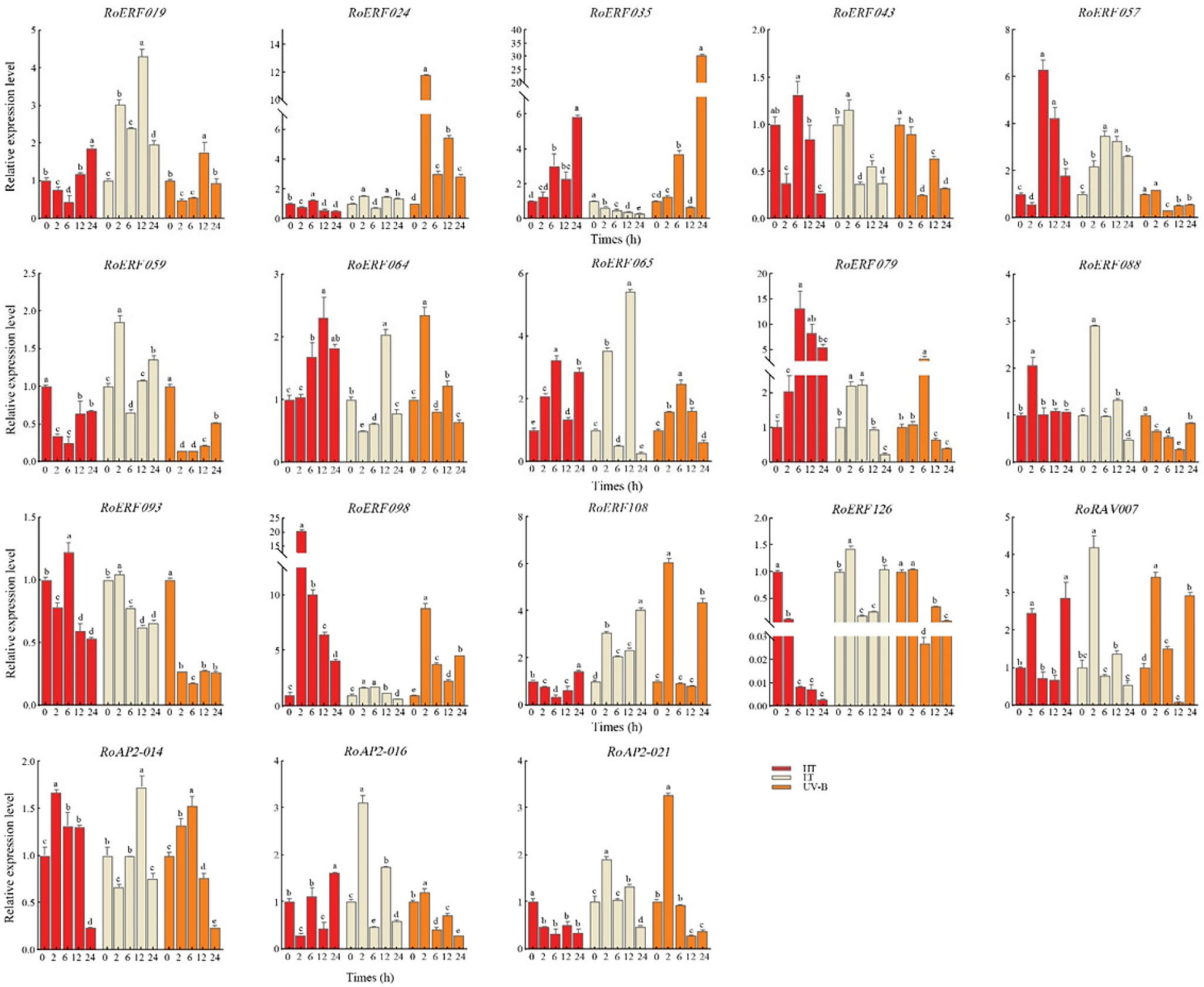
qRT‐PCR validation of some RoAP2/ERF (where AP2/ERF is APETALA2/ethylene‐responsive factor) genes responding to High temperature (HT), Low temperature (LT) and UV‐B. Error bars represent the standard deviation from three biological replicates. Lowercase letters (a, b, c, and d) above the bar graphs indicate significant differences (*p* < 0.05).

RT‐qPCR analysis of 18 genes largely confirmed the RNA‐Seq results (Figure 6). All genes except *RoERF079*, *RoERF088*, and *RoAP02‐14* showed expression patterns consistent with RNA‐Seq at 12 h. *RoERF079* and *RoERF098* showed the strongest induction under HT, reaching 13‐ and 20‐fold increases relative to controls. All genes responded to UV‐B, with *RoERF035* showing the highest induction (∼30‐fold at 24 h).

### 3.7 Expression profiles of the RoAP2/ERF gene family under exogenous hormone treatments

**FIGURE 7:**
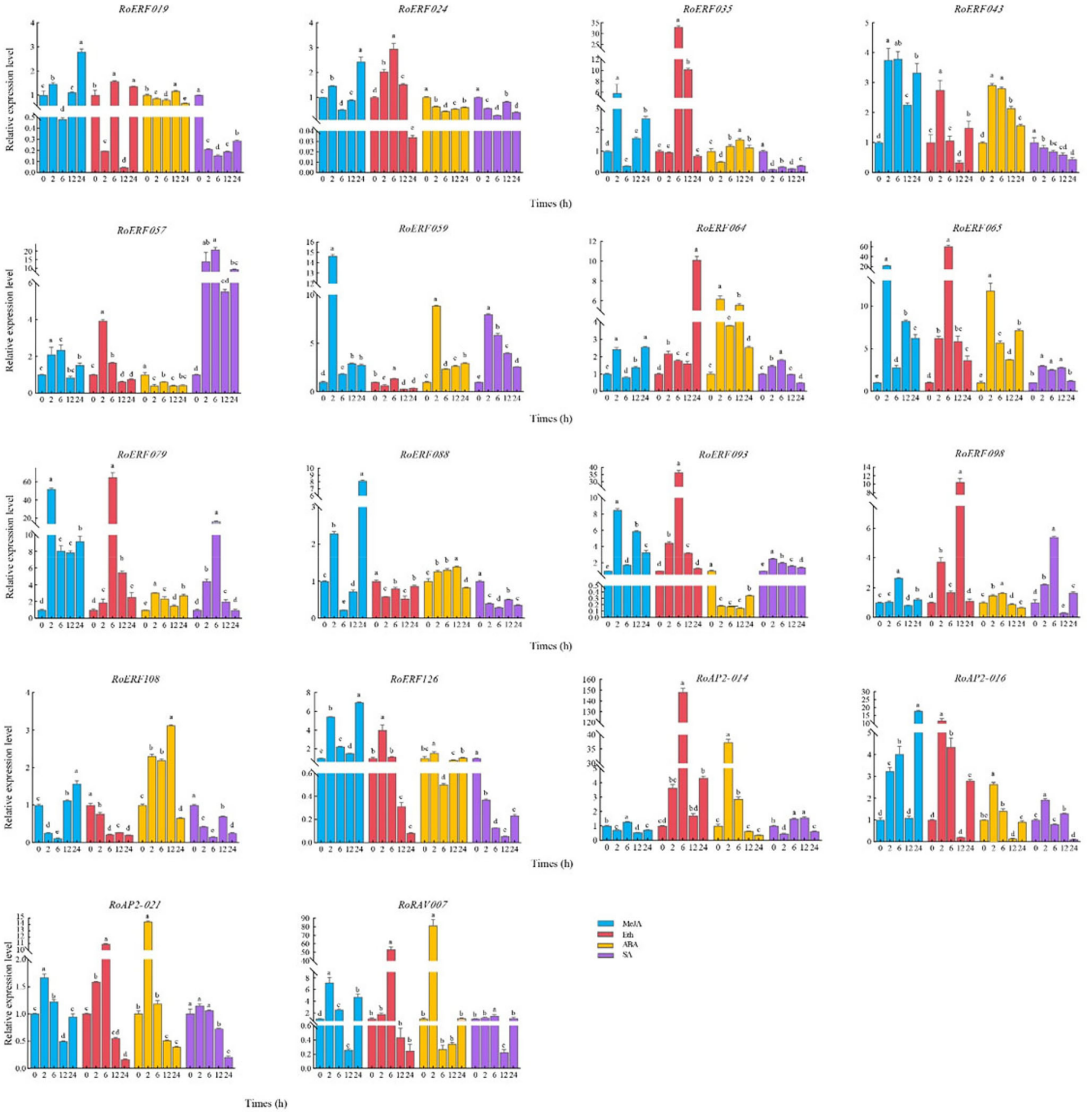
Expression profiles of the RoAP2/ERF (where AP2/ERF is APETALA2/ethylene‐responsive factor) gene family under exogenous hormone treatments (methyl jasmonate [MeJA], Eth, abscisic acid (ABA), and salicylic acid [SA]). Error bars represent the standard deviation from three biological replicates. Lowercase letters (a, b, c, and d) above the bar graphs indicate significant differences (*p* < 0.05).

To further elucidate the transcriptional responses of RoAP2/ERF genes to exogenous phytohormones, RNA‐seq datasets from MeJA‐ and Eth‐treated seedlings were analyzed (Figure S6; Tables S17–S18). More than half of the RoAP2/ERF genes showed significant expression changes under both treatments. RT‐qPCR analysis of 18 representative genes confirmed these patterns (Figure 7). Under MeJA treatment, nine genes were upregulated and nine downregulated at 12 h, with *RoERF059*, *RoERF065*, and *RoERF079* showing the strongest induction, peaking at 2 h (14.65‐, 22.41‐, and 52.25‐fold, respectively). Under Eth treatment, 13 genes were upregulated at 2 h, and most genes peaked at 6 h, with *RoERF065*, *RoERF079*, and *RoRAV7* exceeding 50‐fold induction. Abscisic acid (ABA) and salicylic acid (SA) treatments were further analyzed. Eight genes peaked at 2 h under ABA, with *RoAP02‐14* and *RoRAV07* exceeding 30‐fold induction, whereas *RoERF057* and *RoERF079* showed the strongest responses to SA at 6 h. *RoERF065* and *RoERF079* were consistently induced by all four hormones, suggesting roles as central regulators in multiple signaling pathways.

### 3.8 Co‐expression correlation analysis between RoAP2/ERF genes and key enzymes in the anthraquinone biosynthetic pathway

**FIGURE 8:**
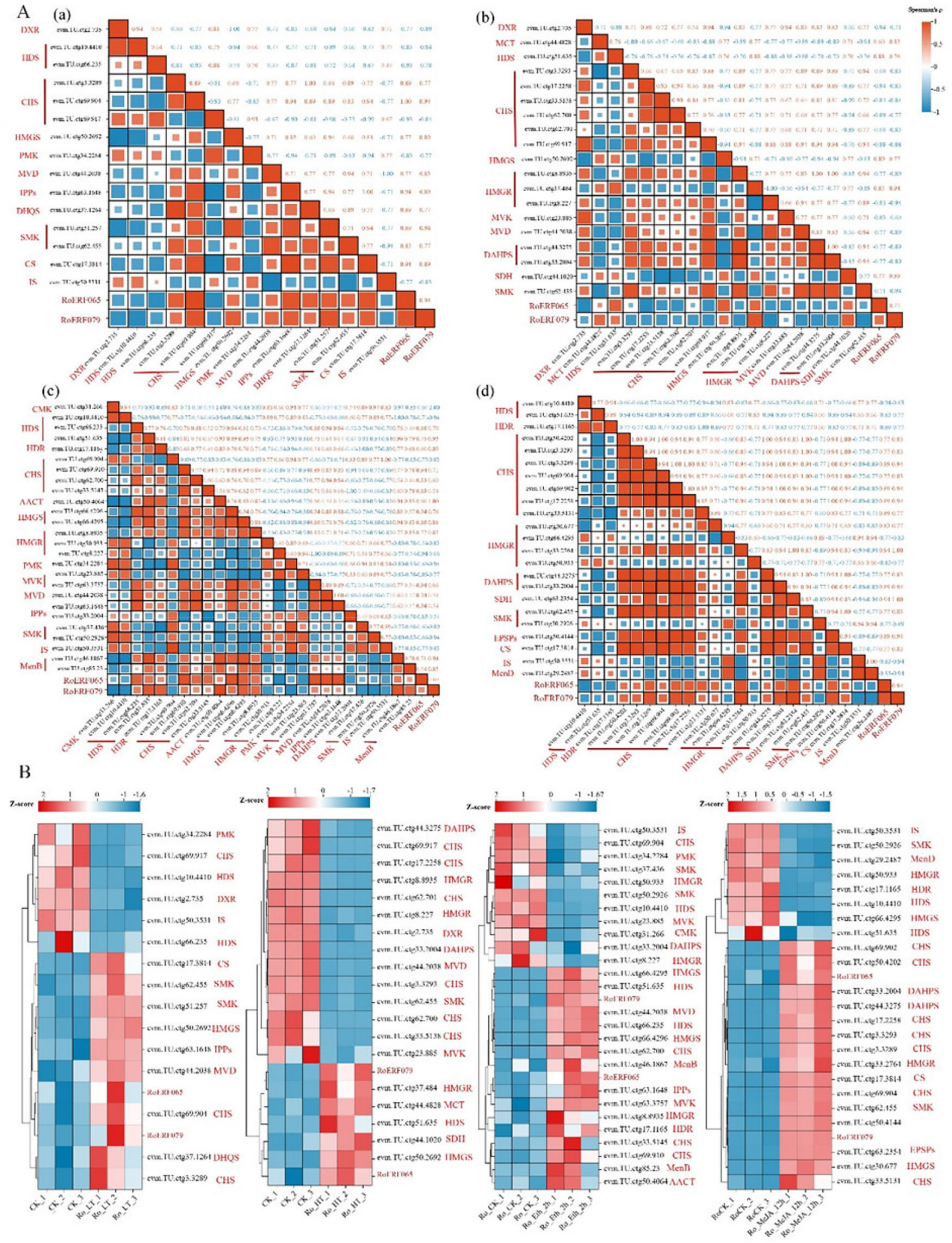
Gene expression analysis between *RoERF065*, *RoERF079* and structural enzyme genes under different treatments. (A) Gene expression correlation matrix under different treatments. Gene pairs with Spearman's *ρ* > 0.8 and *p* < 0.05 were defined as strongly correlated gene pairs. (a) Low temperature; (b) High temperature; (c) Ethephon; (d) Methyl jasmonate. (B) Expression pattern analysis of strongly correlated gene pairs.

A total of 74 putative structural enzyme genes involved in anthraquinone biosynthesis were identified, including members of the MVA, MEP, shikimate, and polyketide pathways (Table S5). Co‐expression analysis under low‐temperature, high‐temperature, Eth, and MeJA treatments revealed distinct treatment‐specific patterns (Figure 8; Tables S19–S25). Low‐temperature treatment yielded the fewest strong correlations, whereas HT predominantly resulted in negative correlations, with only limited genes across pathways associated with *RoERF065* and *RoERF079*. Eth treatment showed the highest number of strongly correlated genes with both TFs. Under MeJA treatment, *RoERF065* and *RoERF079* were strongly co‐expressed and positively correlated with multiple key structural genes, which were also mutually correlated. Promoter analysis revealed enrichment of low‐temperature‐ and MeJA‐responsive elements in these genes, while high‐temperature‐ and ethylene‐responsive elements were absent (Figure S7; Table S26). Collectively, these results indicate that *RoERF065* and *RoERF079* are more likely to participate in the regulation of anthraquinone biosynthesis through low‐temperature and MeJA signaling, with potential synergistic effects under MeJA treatment.

### 3.9 Subcellular localization and transcription activity of RoERF065 and RoERF079

**FIGURE 9:**
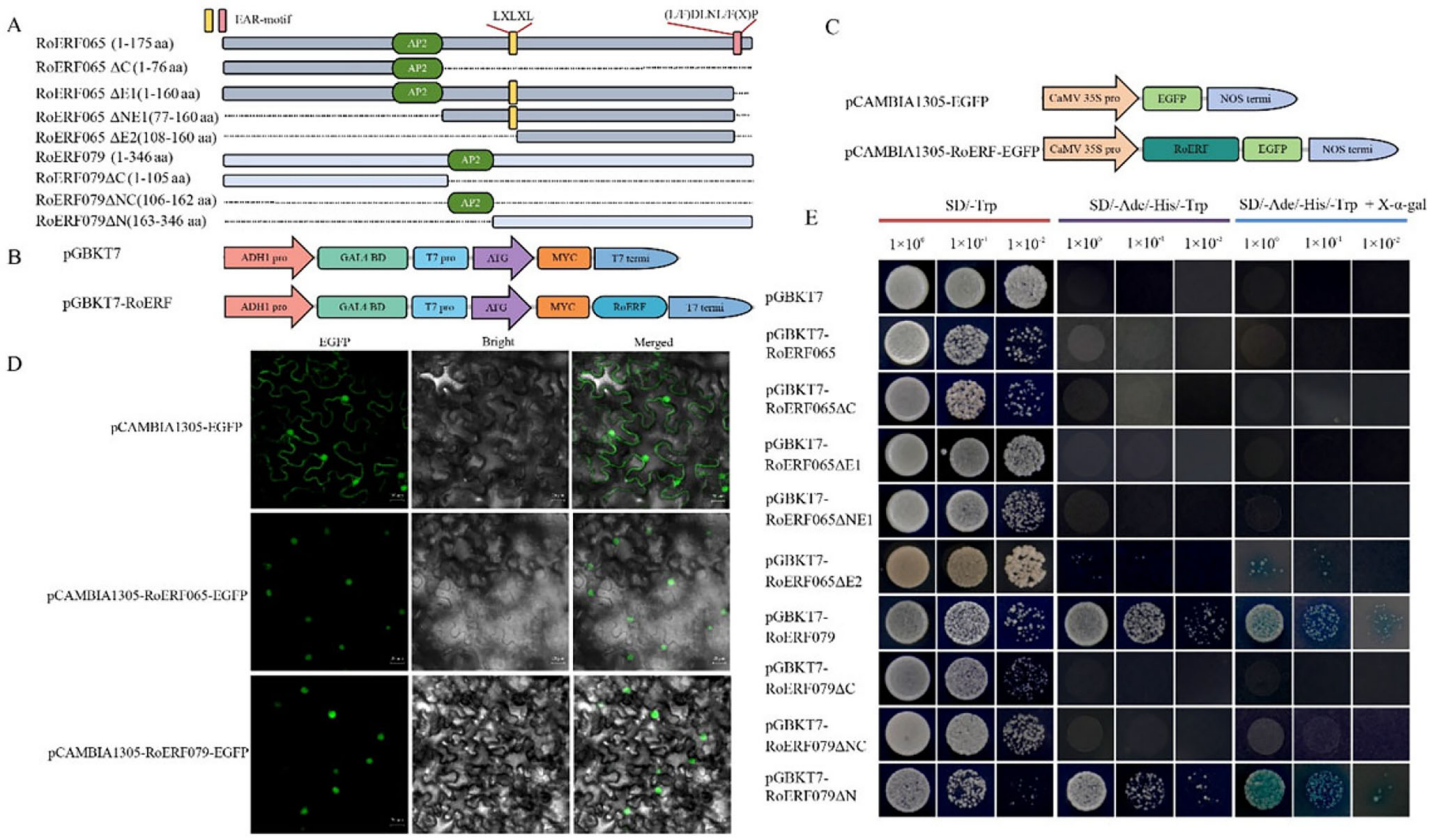
Subcellular localization and transcription activity of RoERF065 and RoERF079. (A) Truncated fragments of RoERF065 and RoERF079 amino acid sequences, with their functional motifs. (B) Schematic diagram of recombinant vectors for yeast one‐hybrid assay. (C) Schematic diagram of recombinant vectors for subcellular localization assay. (D) GFP fluorescence of RoERF065‐EGFP and RoERF079‐EGFP. EGFP indicates fluorescence under dark field; Bright field shows bright‐field images; Merged displays the overlay of both. Scale bar = 20 µm. (E) Transcriptional self‐activation activity of RoERF065, RoERF079, and their truncated fragments in yeast. Yeast strain Y2H Gold was cultured on SD/‐Trp, SD/‐Ade/‐His/‐Trp, and SD/‐Ade/‐His/‐Trp + X‐α‐Gal media. Each colony image is presented as a standardized square of 1 cm side length.

*RoERF079* was predominantly expressed in roots, whereas *RoERF065* expression in roots and rhizomes increased with plant age. Both genes responded to abiotic stresses (heat, cold, and UV‐B) and hormone treatments (MeJA, Eth, ABA, and SA), suggesting roles in development, stress responses, and hormone signaling. RoERF065 and RoERF079 were predicted to localize to the nucleus (Table S8). Subcellular localization was validated by transient expression of EGFP fusion constructs in tobacco epidermal cells. Both RoERF065 and RoERF079 localized exclusively to the nucleus (Figure 9D). These results indicate that RoERF065 and RoERF079 are nuclear‐localized proteins.

Transcriptional activity was assessed using full‐length and truncated constructs fused to the pGBKT7 DNA‐binding domain (Figure 9C). The results showed that all yeast transformants grew normally on SD/‐Trp medium. Full‐length RoERF079 and its N‐terminal fragment activated reporter genes and supported growth on selective media (Figure 9E). The C‐terminal truncation abolished growth and reporter activation. These results indicate that RoERF079 has transcriptional activation activity dependent on its C‐terminus. In contrast, RoERF065 showed no transcriptional activation activity. Sequence analysis revealed that RoERF065 contains two EAR repressor motifs [LXLXL and (L/F)DLNL/F(X)] at its C‐terminus (Figure S8). Only the truncated fragment lacking both EAR domains exhibited very weak transcriptional activation activity.

## 4 DISSCUSSION

The AP2/ERF superfamily plays key roles in plant development, stress responses, and hormone signaling. Understanding its regulatory mechanisms is essential for elucidating stress tolerance and secondary metabolism in medicinal plants. Although characterized in *Isatis indigotica * (Xiao et al., 2023) and *Zingiber officinale* (Xing et al., 2021), the AP2/ERF family remains poorly understood in *R. officinale*. A total of 167 AP2/ERF genes were identified in *R. officinale*, with the ERF subfamily comprising the largest proportion (79.6%). This distribution is consistent with *Isatis tinctoria* (Xiao et al., 2023) and *Zingiber officinale* (Xing et al., 2021), indicating conserved expansion and retention of ERF genes during evolution. Most RoAP2/ERF proteins are unstable and localize to the nucleus or nucleocytoplasm, with secondary structures dominated by α‐helices and random coils—features compatible with transcriptional regulation.

All RoAP2/ERF proteins contain at least one AP2 domain (50–70 aa) with conserved motifs (YRG, RAYD, and (Y/W/H)LG). Most RoERF, RoDREB, and RoRAV members contain the WLG motif, whereas AP2 and Soloist subfamilies contain YLG and HLG motifs, respectively (M. Zhang et al., 2019). The absence of the HLG motif in the Soloist subfamily, also observed in gymnosperms such as *Taxus chinensis* and *Picea abies* (M. Zhang et al., 2019), suggests evolutionary relaxation of this motif in certain taxa. Residue differences at positions 14 and 19 between ERF and DREB are largely conserved, supporting their roles in DNA‐binding specificity (Ohme‐Takagi & Shinshi, 1995). In addition, members such as RoERF123 and RoERF061 are classified into the AP2 subfamily for their C‐terminal AP2‐like domain (no YLG motif), whereas RoERF111 and RoERF112 belong to the RAV subfamily for their B3‐like domain—this pattern is also reported in *Fagopyrum tataricum* (M. Liu et al., 2019) and ramie (Qiu et al., 2022).

Motifs outside the AP2 domain further expand regulatory functions. Some RoERF proteins in groups A5 and B1 contain a C‐terminal (L/F)DLNL/F(X)P motif—a hybrid of canonical EAR motifs associated with transcriptional repression (L. X. Li, Wei, et al., 2021). The EAR motif is also found in other protein families, such as R2R3‐MYB (Leng et al., 2025) and bHLH (W. Wang, He, et al., 2025). A conserved DCDSSS motif was identified at the N‐terminus of group B1 RoERFs, co‐occurring with EAR motifs across species, indicating evolutionary conservation. Gene structure analysis revealed that ERF and RAV members are predominantly intronless, whereas AP2 genes contain 2–9 introns, consistent with that observed in *F. tataricum* (M. Liu et al., 2019), *M. rubra* (Y. Liu et al., 2024), and ginger (Xiao et al., 2023). Multi‐AP2 domain proteins (e.g., RoAP2‐19, RoAP2‐20) suggest lineage‐specific expansion beyond canonical structures. Gene duplication (segmental and tandem) contributed to family expansion, with purifying selection maintaining functional conservation. High collinearity between *R. officinale* and *R. palmatum* further supports evolutionary conservation. The abundance of diverse cis‐elements provides a regulatory basis for multifunctional roles.

Secondary metabolite accumulation is closely associated with tissue‐specific gene expression. For example, schisandrin B accumulation in *Schisandra chinensis* fruits is linked to genes such as *PAL1* and *C4H‐2*, whereas in roots, it correlates with *CCR5* and *SDH4* (Sun et al., 2023). In *R. officinale*, key anthraquinone biosynthesis genes such as *CHS* and *UGT73* show root‐enriched expression (H. Zhang, He, et al., 2024), consistent with the higher anthraquinone accumulation in roots observed across the *Rheum* genus (S. Zhao et al., 2024). Our tissue‐specific expression analysis revealed that Cluster 2 RoAP2/ERF genes were enriched in 3–4‐year‐old roots, while Cluster 5 genes exhibited age‐dependent upregulation in roots and rhizomes—a pattern paralleling the developmental accumulation of anthraquinones (Y. M. Li et al., 2025; S. Zhao et al., 2024). This correlation suggests that certain RoAP2/ERF genes may coordinately regulate organ development and secondary metabolism. Single‐cell transcriptomics offers a promising avenue to dissect spatial regulation. Recent studies have demonstrated that individual TFs can act as cell‐type‐specific hubs linking development to metabolic pathways (X. B. Wang, Ge, et al., 2025). In *R. tanguticum*, free and bound anthraquinones exhibit distinct spatial distributions, with free forms enriched in the bark and bound forms in the core (S. Zhao et al., 2024). The root‐enriched expression of RoAP2/ERF genes suggests potential cell‐type‐specific roles in anthraquinone biosynthesis. Future single‐cell analyses may further resolve these spatial regulatory mechanisms.

Environmental factors regulate TFs through hormone signaling pathways. Previous studies show that *PtrERF110* overexpression in citrus promotes the biosynthesis of sugars and sterols while enhancing cold tolerance (Khan et al., 2024). *RoERF064, RoERF065, RoERF079*, and *RoRAV07* showed strong responses to temperature and UV‐B stress, suggesting roles in stress adaptation and metabolic regulation. RoAP2/ERF genes also exhibited diverse responses to MeJA, SA, ABA, and ET, with *RoERF065* and *RoERF079* induced by all treatments, indicating potential roles in hormone signaling crosstalk. These findings support the central role of AP2/ERF genes in integrating environmental and hormonal signals to regulate stress responses and secondary metabolism.

RoERF065 and RoERF079 displayed root‐preferential expression and were strongly co‐expressed with key anthraquinone biosynthetic genes under MeJA treatment. Both proteins localized to the nucleus, consistent with TF function. RoERF079 exhibited strong transcriptional activation dependent on its C‐terminal domain, whereas RoERF065 contains EAR repression motifs, suggesting a repressive role. EAR‐containing TFs typically regulate target genes via co‐repressor recruitment and chromatin modification (Ni et al., 2023). Together, these results suggest that RoERF065 and RoERF079 may function as opposing regulators coordinating stress responses and anthraquinone biosynthesis in *R. officinale*.

Despite providing the first genome‑wide characterization of the AP2/ERF family in *R. officinale*, this study has several limitations that warrant consideration. First, direct genetic evidence linking RoERF065 or RoERF079 to anthraquinone biosynthesis remains absent; future investigations employing virus‑induced gene silencing (VIGS) or CRISPR/Cas9‑mediated knockout, coupled with targeted metabolite profiling, are necessary to establish causal relationships. Second, bulk tissue expression analysis cannot resolve cell‑type‑specific regulatory roles; single‑cell transcriptomics offers a promising avenue to identify which root cell layers express specific RoAP2/ERF genes and how they correlate with anthraquinone compartmentation. Addressing these limitations will substantially advance our understanding of AP2/ERF‑mediated regulation of growth and secondary metabolism in this medicinal species.

## 5 CONCLUSION

This study systematically characterized the composition, structure, and evolution of the AP2/ERF family in *R. officinale*. Transcriptome analysis revealed tissue‐specific expression and stress/hormone responsiveness: Cluster 2 genes were highly expressed in 3–4‐year‐old roots, while Cluster 5 genes exhibited growth‐dependent upregulation in roots and rhizomes, consistent with anthraquinone accumulation. Based on these expression patterns, RoERF065 (Cluster 2) and RoERF079 (Cluster 5) were selected for functional validation. RoERF065 was identified as a dual EAR motif‐mediated transcriptional repressor, whereas RoERF079 functioned as a C‐terminal‐dependent activator, suggesting roles in regulating root and rhizome development and secondary metabolism. This study provides a foundation for understanding the molecular mechanisms of anthraquinone biosynthesis, identifies candidate genes for molecular breeding, and offers targets for future research.

## AUTHOR CONTRIBUTIONS

**Jing Tang**: Investigation; visualization; writing—original draft. **Gao‐chang Cui**: Data curation; investigation; visualization. **Heng Pan**: Conceptualization. **Hui Lu**: Conceptualization. **Yi‐min Li**: Methodology. **Feng Yan**: Formal analysis. **Jing Gao**: Data curation. **Liang Peng**: Data curation. **Xiao‐chen Hu**: Supervision; writing—review and editing. **Gang Zhang**: Supervision; writing—review and editing.

## CONFLICT OF INTEREST STATEMENT

The authors declare no conflicts of interest.

## Supporting information




**Table S1** Protein sequences of the AP2/ERF family from Arabidopsis thaliana and Rheum officinale used for phylogenetic analysis.
**Table S2** Concentrations of exogenous hormones and stress treatment conditions.
**Table S3** Sequence information of RT‐qPCR primers for selected genes from the RoAP2/ERF family.
**Table S4** ID numbers and protein sequences of the enzymes in the NR database.
**Table S5** Sequence information of key enzymes in the anthraquinone synthesis pathway from R. officinale.
**Table S6** Primers used for subcellular localization analysis in this stud.
**Table S7** Primers used for transcriptional activity analysis in this study.
**Table S8** Characteristics of the identified RoAP2/ERF family genes and their encoded proteins.
**Table S9** Segmental and tandem duplicated gene pairs in the RoAP2/ERF family and their Ka/Ks values.
**Table S10** List of syntenic gene pairs of the AP2/ERF family between R. officinale and Oryza sativa.
**Table S11** List of syntenic gene pairs of the AP2/ERF family between R. officinale and Rheum tanguticum.
**Table S12** List of syntenic gene pairs of the AP2/ERF family between R. officinale and Rheum palmatum.
**Table S13** List of syntenic gene pairs of the AP2/ERF family between R. officinale and Rheum nobile.
**Table S14** Analysis of cis‐elements in the promoter regions of RoAP2/ERF family genes.
**Table S15** Expression patterns of RoAP2/ERF family genes in various tissues of 2‐, 3‐, and 4‐year‐old R. officinale plants.
**Table S16** Expression patterns of RoAP2/ERF family genes in response to heat and cold stress.
**Table S17** Expression patterns of RoAP2/ERF family genes in response to ethylene.
**Table S18** Expression patterns of RoAP2/ERF family genes in response to methyl jasmonate.
**Table S19** Expression levels of genes encoding key enzymes in the anthraquinone synthesis pathway under heat and cold stress conditions.
**Table S20** Expression levels of genes encoding key enzymes in the anthraquinone synthesis pathway under ethylene treatment.
**Table S21** Expression levels of genes encoding key enzymes in the anthraquinone synthesis pathway under methyl jasmonat treatment.
**Table S22** Correlation analysis between expression levels of RoAP2/ERF genes and key anthraquinone biosynthetic enzyme genes under cold stress.
**Table S23** Correlation analysis between expression levels of RoAP2/ERF genes and key anthraquinone biosynthetic enzyme genes under heat stress.
**Table S24** Correlation analysis between expression levels of RoAP2/ERF genes and key anthraquinone biosynthetic enzyme genes under ethylene treatment.
**Table S25** Correlation analysis between expression levels of RoAP2/ERF genes and key anthraquinone biosynthetic enzyme genes under methyl jasmonat treatment.
**Table S26** Analysis of cis‐elements in the promoter regions of anthraquinone biosynthetic pathway genes that are significantly correlated with the expression of RoERF065 and RoERF079.

**Figure S1** Conserved domains of AP2/ERF family members in Rheum officinale.
**Figure S2** Motif analysis of the RoAP2/ERF family members.
**Figure S3** Motifs of the RoAP2/ERF family members.
**Figure S4** Gene structure of the RoAP2/ERF family members. CDS, represent coding sequences. UTR, untranslated region. Gray lines represent introns.
**Figure S5** Expression patterns of the RoAP2/ERF family members in various plant tissues and in response to abiotic stress. (A) Expression patterns of the RoAP2/ERF family members in various tissues of 2‐, 3‐, and 4‐year‐old R. officinale. The FPKM matrix of all expressed genes underwent row‐wise Z‐score normalization. Color intensity indicates Z‐score values (blue: downregulated, red:upregulated). Ro_1_L, Ro_2_L, and Ro_3_L denote leaves; Ro_1_R, Ro_2_R, and Ro_3_R denote roots; Ro_1_RH, Ro_2_RH, and Ro_3_RH denote rhizomes—with the numerals “1”, “2”, and “3” in these identifiers correspondin g to two‐year‐old, three‐year‐old, and four‐year‐old plants, respectively. (B) Expression patterns of the RoAP2/ERF family members under 40°C (HT) and 4°C (LT). The heatmap was generated based on FPKM values. Color intensity indicates FPKM values (blue: low expression, red: high expression).
**Figure S6** Expression profiles of the RoAP2/ERF family members under exogenous hormone treatments (Eth, Ethephon; MeJA, Methyl jasmonate). The expression values of each gene are represented by a color scale of log_2_ (fold change), where red and blue indicate upregulation and downregulation, respectively. Detailed log2 (fold change) and FDR‐adjusted p are provided in Tables S17 and S18.
**Figure S7** Cis‐acting element analysis of structural enzyme genes in the anthraquinone biosynthetic pathway that show strong expression correlation with RoERF065 or RoERF079.
**Figure S8** Distribution of the amino acid sequence and functional domains of RoERF065.

## Data Availability

Data will be made available on request.
