# Correction to “Genome‐wide analysis of theAP2/ERF gene family in *Rheum officinale* Baill.: Evolution and expression profiling during plant development, abiotic stresses, and exogenous hormone responses”

**DOI:** 10.1002/tpg2.70260

**Published:** 2026-05-29

Tang, J., Cui, G.‐C., Pan, H., Lu, H., Li, Y.‐M., Yan, F., Gao, J., Peng, L., Hu, X.‐C., & Zhang, G. (2026). Genome‐wide analysis of theAP2/ERF gene family in *Rheum officinale* Baill.: Evolution and expression profiling during plant development, abiotic stresses, and exogenous hormone responses. *The Plant Genome*, *19*, e70248. https://doi.org/10.1002/tpg2.70248

The email addresses of the two co‐corresponding authors, Xiao‐chen Hu and Gang Zhang, were inadvertently swapped in the corresponding author information. The original text appeared as “Xiao‐chen Hu and Gang Zhang, Key Laboratory for Research and Development of “Qin Medicine” of Shaanxi Administration of Traditional Chinese Medicine, Shaanxi University of Chinese Medicine, Xianyang, 712046, China. E‐mail: jay_gumling2025@163.com and huxc8918@163.com.” This has now been corrected to “Xiao‐chen Hu and Gang Zhang, Key Laboratory for Research and Development of “Qin Medicine” of Shaanxi Administration of Traditional Chinese Medicine, Shaanxi University of Chinese Medicine, Xianyang, 712046, China. E‐mail: huxc8918@163.com and jay_gumling2025@163.com.”

We apologize for this error.

