# Supplementary material for: Genome‐wide analysis of the AP2/ERF gene family in Rheum officinale Baill.: Evolution and expression profiling during plant development, abiotic stresses, and exogenous hormone responses
Source: Plant Genome. 2026 May 11;19(2):e70248. doi: 10.1002/tpg2.70248 (PMC13158899; doi:10.1002/tpg2.70248)
Supplement: Supplementary file 1 [file TPG2-19-e70248-s001.docx]

Supplemental Material

Supplemental Material includes Supplemental Figures (S1–S8) and Supplemental Tables (S1–S26).

Figure S1 Conserved domains of AP2/ERF family members in Rheum officinale.

Figure S2 Motif analysis of the RoAP2/ERF family members.

Figure S3 Motifs of the RoAP2/ERF family members.

Figure S4 Gene structure of the RoAP2/ERF family members. CDS, represent coding sequences. UTR, untranslated region. Gray lines represent introns.

Figure S5 Expression patterns of the RoAP2/ERF family members in various plant tissues and in response to abiotic stress. (A) Expression patterns of the RoAP2/ERF family members in various tissues of 2-, 3-, and 4-year-old R. officinale. The FPKM matrix of all expressed genes underwent row-wise Z-score normalization. Color intensity indicates Z-score values (blue: downregulated, red:upregulated). Ro_1_L, Ro_2_L, and Ro_3_L denote leaves; Ro_1_R, Ro_2_R, and Ro_3_R denote roots; Ro_1_RH, Ro_2_RH, and Ro_3_RH denote rhizomes—with the numerals “1”, “2”, and “3” in these identifiers correspondin g to two-year-old, three-year-old, and four-year-old plants, respectively. (B) Expression patterns of the RoAP2/ERF family members under 40 ℃ (HT) and 4 ℃ (LT). The heatmap was generated based on FPKM values. Color intensity indicates FPKM values (blue: low expression, red: high expression).

Figure S6 Expression profiles of the RoAP2/ERF family members under exogenous hormone treatments (Eth, Ethephon; MeJA, Methyl jasmonate). The expression values of each gene are represented by a color scale of log2 (fold change), where red and blue indicate upregulation and downregulation, respectively. Detailed log2 (fold change) and FDR-adjusted p are provided in Supplementary Table S17 and Supplementary Table S18

Figure S7 Cis-acting element analysis of structural enzyme genes in the anthraquinone biosynthetic pathway that show strong expression correlation with RoERF065 or RoERF079

Figure S8 Distribution of the amino acid sequence and functional domains of RoERF065.

Table S1 Protein sequences of the AP2/ERF family from Arabidopsis thaliana and Rheum officinale used for phylogenetic analysis.

Table S2 Concentrations of exogenous hormones and stress treatment conditions.

Table S3 Sequence information of RT-qPCR primers for selected genes from the RoAP2/ERF family.

Table S4 ID numbers and protein sequences of the enzymes in the NR database

Table S5 Sequence information of key enzymes in the anthraquinone synthesis pathway from R. officinale.

Table S6 Primers used for subcellular localization analysis in this stud.

Table S7 Primers used for transcriptional activity analysis in this study.

Table S8 Characteristics of the identified RoAP2/ERF family genes and their encoded proteins.

Table S9 Segmental and tandem duplicated gene pairs in the RoAP2/ERF family and their Ka/Ks values.

Table S10 List of syntenic gene pairs of the AP2/ERF family between R. officinale and Oryza sativa.

Table S11 List of syntenic gene pairs of the AP2/ERF family between R. officinale and Rheum tanguticum.

Table S12 List of syntenic gene pairs of the AP2/ERF family between R. officinale and Rheum palmatum.

Table S13 List of syntenic gene pairs of the AP2/ERF family between R. officinale and Rheum nobile.

Table S14 Analysis of cis-elements in the promoter regions of RoAP2/ERF family genes.

Table S15 Expression patterns of RoAP2/ERF family genes in various tissues of 2-, 3-, and 4-year-old R. officinale plants.

Table S16 Expression patterns of RoAP2/ERF family genes in response to heat and cold stress.

Table S17 Expression patterns of RoAP2/ERF family genes in response to ethylene.

Table S18 Expression patterns of RoAP2/ERF family genes in response to methyl jasmonate.

Table S19 Expression levels of genes encoding key enzymes in the anthraquinone synthesis pathway under heat and cold stress conditions.

Table S20 Expression levels of genes encoding key enzymes in the anthraquinone synthesis pathway under ethylene treatment.

Table S21 Expression levels of genes encoding key enzymes in the anthraquinone synthesis pathway under methyl jasmonat treatment.

Table S22 Correlation analysis between expression levels of RoAP2/ERF genes and key anthraquinone biosynthetic enzyme genes under cold stress.

Table S23 Correlation analysis between expression levels of RoAP2/ERF genes and key anthraquinone biosynthetic enzyme genes under heat stress.

Table S24 Correlation analysis between expression levels of RoAP2/ERF genes and key anthraquinone biosynthetic enzyme genes under ethylene treatment.

Table S25 Correlation analysis between expression levels of RoAP2/ERF genes and key anthraquinone biosynthetic enzyme genes under methyl jasmonat treatment.

Table S26 Analysis of cis-elements in the promoter regions of anthraquinone biosynthetic pathway genes that are significantly correlated with the expression of RoERF065 and RoERF079.
